# Crowd behavior representation: an attribute-based approach

**DOI:** 10.1186/s40064-016-2786-0

**Published:** 2016-07-26

**Authors:** Hamidreza Rabiee, Javad Haddadnia, Hossein Mousavi

**Affiliations:** 1 Electrical Engineering Department, Hakim Sabzevari University, Sabzevar, Iran; 2 Biomedical Engineering Department, Hakim Sabzevari University, Sabzevar, Iran; 3 Pattern Analysis and Computer Vision Department (PAVIS), Istituto Italiano di Tecnologia, Genoa, Italy

**Keywords:** Crowd behavior, Crowd emotions, Mid-level representation, Low-level features

## Abstract

In crowd behavior studies, a model of crowd behavior needs to be trained using the information extracted from video sequences. Most of the previous methods are based on low-level visual features because there are only crowd behavior labels available as ground-truth information in crowd datasets. However, there is a huge semantic gap between low-level motion/appearance features and high-level concept of crowd behaviors. In this paper, we tackle the problem by introducing an attribute-based scheme. While similar strategies have been employed for action and object recognition, to the best of our knowledge, for the first time it is shown that the crowd emotions can be used as attributes for crowd behavior understanding. We explore the idea of training a set of emotion-based classifiers, which can subsequently be used to indicate the crowd motion. In this scheme, we collect a large dataset of video clips and provide them with both annotations of “crowd behaviors” and “crowd emotions”. We test the proposed emotion based crowd representation methods on our dataset. The obtained promising results demonstrate that the crowd emotions enable the construction of more descriptive models for crowd behaviors. We aim at publishing the dataset with the article, to be used as a benchmark for the communities.

## Background

Learning-based methods for human behavior recognition have been the subject of various studies over the last years. The behavior analysis frameworks are regularly built on patterns of low-level motion/appearance features, e.g. HOG, HOF, HOT, etc. (Chen et al. 2007; Kratz and Nishino 2009, 2010; Krausz and Bauckhage 2011, 2012; Li et al. 2014; Mahadevan et al. 2010; Mehran et al. 2009; Raghavendra et al. 2011; Rodriguez et al. 2011; Roggen et al. 2011; Saxena et al. 2008; Solmaz et al. 2012; Su et al. 2013; Wang et al. 2012; Zhang et al. 2012). These features are directly related to behavior types (such as panic, fight, neutral, etc.) using modern machine learning techniques, e.g. support vector machines. For instance, in Krausz and Bauckhage (2011, 2012), optical flow histograms are used to demonstrate the global motion in a crowded scene. They derive the histogram of the optical flow and extract some statistics from it to model human behaviors. Then, a set of simple heuristic rules are used to detect specific dangerous crowd behaviors. More advanced techniques, on the other hand, introduce models extracted from fluid dynamics or other physics laws to model a crowd as a group of moving particles.

Together with Social Force Models (SFM), it was likely to explain the behavior of a crowd as the result of interaction of individuals (Mehran et al. 2009; Raghavendra et al. 2011). In Mehran et al. (2009), for example, the SFM is applied to detect global abnormalities and estimate local abnormalities by detecting focus regions in the current frame. On the other hand, several approaches cope with the complexity of a dynamic scene analysis by partitioning a given video in spatial–temporal patches. Kratz and Nishino (2009, 2010) derive spatial–temporal gradients from each pixel of videos. Then, the gradients of a spatio-temporal volume are modeled using spatial–temporal Motion Pattern Models, which are basically 3D Gaussian clusters of gradients. Using dynamic textures, Mahadevan et al. (2010) model the observed motion in each spatial temporal volume, which can be considered as an extension of PCA-based representation. Whereas PCA spaces only model the appearance of a given patch texture, dynamic textures also represent the statistically valid transitions among textures in a patch. Making use of a Mixture of Dynamic Texture (MDT), all the possible dynamic textures are represented and allowing to estimate the probability of a test patch to be abnormal. In this way, it was shown that not only temporal anomalies but also pure appearance anomalies can be detected. In the same work the authors introduced also an interesting definition of spatial saliency based on Mutual Information (Li et al. 2014) between features and foreground/background classes.

**Fig. 1:**
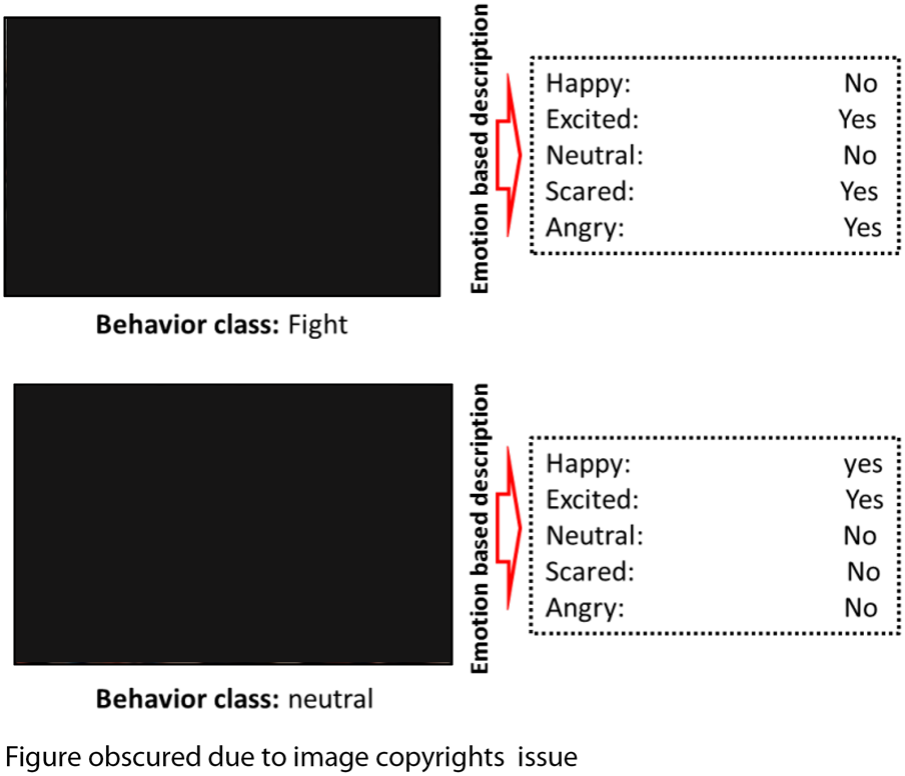
We propose to represent human behaviors by a set of emotions as intermediate representations which can directly be corresponded to the visual specifications. This explains the spatial–temporal evolution of the behavior in a video (e.g., scared, happy, excited, sad, neutral, angry)

**Fig. 2:**
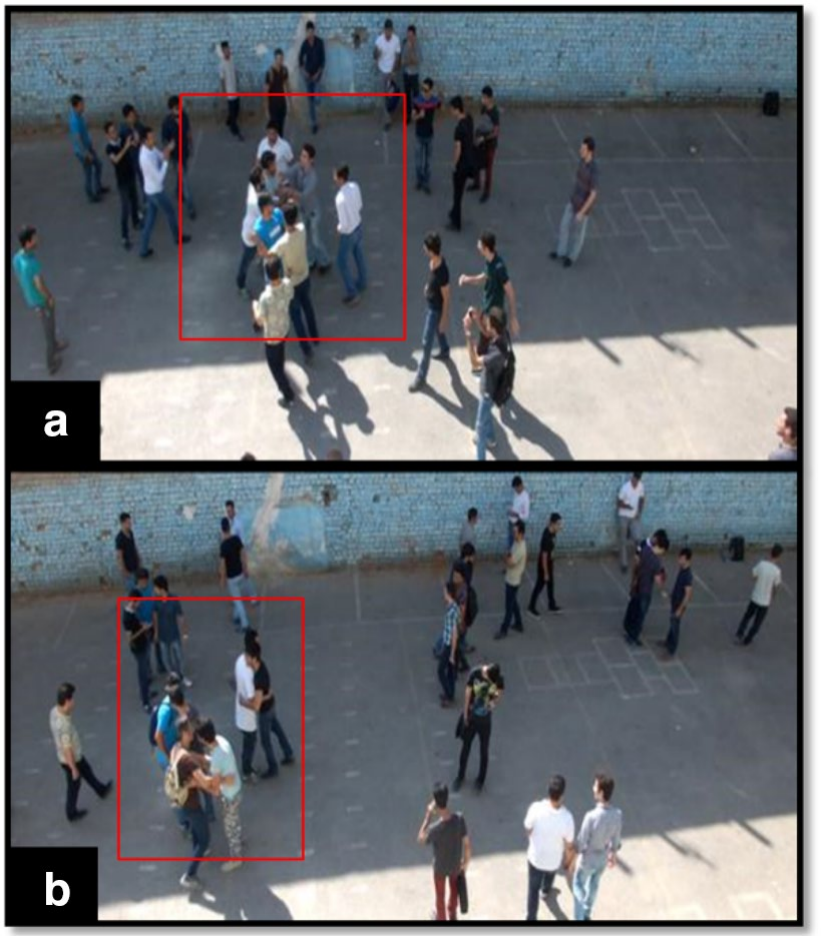
Two sample frames of our dataset. In frame **a** individuals are fighting, while in frame **b** peoples are greeting. Considering only visual low-level features, **a** and **b** are similar, however, they are completely different considering also crowd emotion as high-level semantic representation. Also, in spite of having the “congestion” behavior class in both frames, in frame **a** individuals are “angry”, while in frame **b** individuals are “happy” emotionally

However, in order to achieve better classification accuracy level, aforementioned models need a lot of ground-truth crowd behavior training information for each class, typically hundreds of thousands of sample images for each behavior class to be learned. Therefore, considering high-level semantic concepts such as crowd emotions would be beneficial to represent the crowd behaviors. In different circumstances, emotions belonging to individuals have a significant effect on their behaviors. For example, in a pure low-level feature based behavior recognition framework, individuals who approach each other, and shake hands might be considered as a fighting crowd, whereas they behave normally and are happy emotionally (see Fig. 2). In other words, in terms of classifying crowd behaviors, two groups of individuals who are very similar in aspect of only low-level features, might found to be entirely different by considering crowd emotions as well (see Figs. 1, 2).

Despite the relatively vast literature on emotion recognition for face (Cowie et al. 2001; Ekman 1992; Ekman and Friesen 1977; Ekman and Scherer 1984; Goldman and Sripada 2005; Schuller et al. 2003) and posture (Coulson 2004; Dmello et al. 2008; Mota and Picard 2003), there is just a few works which aim at emotion recognition from crowd motion (Baig et al. 2015; Baig et al. 2014; McHugh and McDonnell 2010), and to the best of our knowledge, there is no work which aims at crowd emotion recognition and behavior types detection in an integrated framework. Lack of publicly available realistic datasets (i.e., with high density crowds, various types of behaviors, etc.) is another constraint. This causes difficulty for researchers to have a reasonable common test bench to compare their works and fairly evaluate the strength and efficiency of their methods in real scenarios.

Inspired by the recent works on attribute-based representation in object/action recognition literature (Farhadi et al. 2009, 2010; Lampert et al. 2009; Liu et al. 2011), we aim at build a framework wherein crowd emotion information are used as a mid-level representation for crowd behavior recognition. The capability of determining behaviors by emotions as kind of attributes in behavior classification is beneficial to recognize not only familiar behaviors but to recognize behavior classes which have never been seen before and there are no training samples available for them.

### Our contribution

The proposed approach aims at exploring how emotion attributes can improve the crowd behavior recognition process. As the first contribution, we created a crowd dataset with both *crowd behavior* and *crowd emotion* annotations. Our dataset includes a large set of video clips annotated with both crowd behavior labels (e.g., “panic”, “fight”, “congestion”, etc.) and crowd emotion labels (e.g., “happy”, “excite”, “angry”, etc.). We evaluated a set of baseline methods on both behavior class detection and emotion recognition, showing that the proposed dataset can be effectively used as a benchmark in the mentioned communities.

As another contribution, we used ground-truth emotion information provided in our dataset as an intermediate layer to recognize behavior classes. We called this method *Emotion*-*based crowd representation*.

The rest of the paper is constructed as follows: a short review on traditional datasets and the characteristics of our proposed dataset is reported in “Crowd behavior dataset” section; the emotion-based crowd representation idea for crowd behavior recognition is presented in “Emotion-based crowd representation” section. In “Experiments” section we test the proposed methods on our dataset and discuss on the achieved results. Finally, in “Conclusion” section, other worth investigation applications are briefly elaborated, and promoted for further research on the proposed dataset.

## Crowd behavior dataset

In this Section, after a brief review on the state-of-the art crowd datasets for the task of crowd behavior analysis, we present our dataset in details.

### Previous datasets

In the past few years, there has been an explosion of research into the analysis of behaviors occur in crowded videos and as a result, several behavior recognition techniques are designed. However, there is still an impressive gap between precision and efficiency of proposed behavior recognition frameworks in research labs and the real worlds. The most important reason is the lack of publicly available standard benchmark datasets with many individuals and frequent behavior scenarios, which forces the majority of algorithms to be tested on non-standard datasets recorded under controlled circumstances. In this work, six most cited crowd dataset namely, UMN (Mehran et al. 2009), UCSD (Mahadevan et al. 2010), CUHK (Wang et al. 2009), PETS2009 (Ferryman et al. 2009), VIF (Hassner et al. 2012) and Rodrigues’s (Rodriguez et al. 2011) are selected and their specifications are analyzed in detail. We also choose some criteria on which crowd datasets can be compared. The evaluation criteria are consisting of: *number of samples, annotation level, crowd density, type of scenarios, Indoor/Outdoor, meta*-*data*.

*Number of samples* is an important characteristic of a dataset. The more recorded videos exist in a dataset, the greater samples are available at training time and the better efficiency is achieved at evaluation time. *Annotation level* is another important criterion of a crowd dataset. It can be characterized as pixel-level, frame-level and video-level, which technically reflects the richness of a dataset. *Crowd* *density* is another important issue in crowd analysis. In a crowded dataset, one expects to see more individuals, which might face more occlusions and clutters. This characteristic makes the task of behavior type recognition harder and more time consuming. *Type of scenarios* is another important characteristic of a dataset which reflects the type of events happening in the videos. Datasets with more frequent types of scenarios are more realistic and can be considered as more reliable benchmarks. The *Indoor/Outdoor* criterion is about the location in which the video sequences have been recorded and as a result, has a peculiar effect on illumination conditions, background clutters, occlusions, etc. Last but not least, *Meta*-*data* is another important feature of a dataset, which we insist on it in this paper. It is also one of the features which make our dataset unique and provide the possibility for researchers to move toward higher-level interpretations of the video sequences. In our dataset, we specifically, introduced “crowd emotion” as meta-data. In Table 1, we describe all aforementioned crowd behavior datasets in terms of the explained features. A common demerit lies in all of the state-of-the-art datasets is the absence of any meta-data as extra annotation, which makes them to potentially rely only on low-level features to discriminate types of behavior classes. The lack of frequent behavior type scenarios, low density of crowd and limited number of video sequences are other limitations in aforementioned datasets.

**Table 1:** Datasets for crowd behavior analysis

Dataset	UMN	UCSD	CUHK	PETS2009	VIF	Rodriguez’s	Our dataset
Number of samples	11 seq	98 seq	2 seq	59 seq	246 seq	520 seq	43,626 clips
Annotation level	Frame	Frame/pixel	Video	Frame	Video	Video	Frame
Density	Semi	Semi	Semi	Semi	High	High	High
Type of scenarios	Panic	Abnormal object	traffic	Panic	Fight	Pedestrian	Multi-category
Indoor/outdoor	Both	Outdoor	Outdoor	Outdoor	Outdoor	Outdoor	Outdoor
Meta-data	No	No	No	No	No	No	Crowd emotion

### Proposed dataset

The proposed dataset includes 31 video clips or 44,000 individual frames with the resolution of 554 × 235. The video clips and frames were recorded at 30 frames per second using a fixed video recorder elevated at a height, viewing individuals moving.

The crowd density was regarded variable, ranging from sparse to very crowded. In each scenario, the pedestrian locations and direction of walking are randomly selected. In order to make scenarios more realistic and applicable, we used some fixed and passing abnormal objects as threats to individuals in several scenes. Those scenarios are video clips with “a suspicious backpack left by an unknown person in the crowd”, “a Motorcycle passing the crowded scene” and “a motorcycle without rider which is left between individuals”.

In our dataset, we have introduced five distinct basic types of crowd behavior classes. Each scenario configuration was sketched in accordance with circumstances typically met in crowding issues. They can be explained as, namely the normal movements of individuals in a crowded scenes (*neutral*), a crowded scene including abnormal objects (*Obstacles* or *abnormal object*), individuals evacuate the scene (*panic*), physical conflict between individuals (*fight*) and two or more individuals gathering together closely (*congestion*).

In order to reach a crowd dataset with pool of various behavior scenarios, we tried to have at least two video clips relating to each behavior class from different field of views and with diverse crowd density.

In psychology, emotion is defined as “a feeling evoked by environmental stimuli or by internal body states” (Bower and Cohen 1982). This can characterize human behavior in terms of actions in the environment or changes in the internal status of the body. Considering basic emotions, we introduce six types of basic crowd emotions in our dataset namely, “Angry”, “Happy”, “Excited”, “Scared”, “Sad” and “Neutral” as behavior class attributes. As aforementioned, the state-of-the-art techniques for emotion recognition mainly rely on the appearance of face/posture and usually fail in case of high density crowd. We, however, target the emotion recognition from a totally different perspective, and utilize the crowd “motion” instead of “appearance” of individuals. This is specifically useful in low-resolution videos and high-density crowd scenes wherein there is more occlusion and clutter (typical case for video surveillance systems).

To alleviate the subjectivity of emotion and elevate the reliability of the emotion labels in the dataset, a group of 5 workers independently annotated the dataset and the final labels have been selected via majority voting (e.g. picking the label with more votes). To insure the consistency between workers, we conducted an agreement study, finding that the overall agreement between workers in selecting the same crowd emotion attributes was about 92 % with a Kappa value of 0.81 and the maximum inconsistency was between two emotion attributes, namely *Happy* and *Excited*, which were confused with each other almost 4 % of the time.

In Table 2, some beneficial information from recorded video clips are presented, which include total number of frames and also number of frames associated with each predefined behavior and emotion class.

**Table 2:** Number of frames corresponding to each behavior and emotion label along with total number of frames available in our dataset

Behavior labels	Number of frames	Emotion labels	Number of frames
Panic	2002	Angry	5915
Fight	4423	Happy	1977
Congestion	2368	Excited	3804
Obstacle	5120	Scared	1975
Neutral	29,713	Sad	1140
		Neutral	28,815
Total	43,626	Total	43,626

Our specified emotion labels as attributes can assist behavior class recognition because they present high-level semantic information which are much richer than pure low-level visual features and might be applied for improving the characterization of behavior classes and providing more descriptive and discriminative framework for the task of crowd behavior classification.

**Fig. 3:**
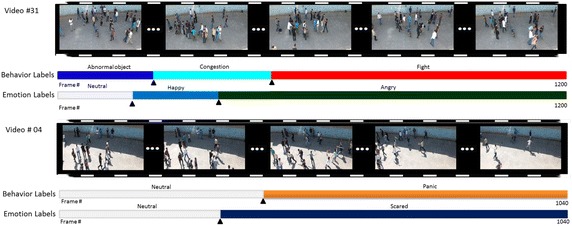
The qualitative results of both emotion and behavior detection for two sample video clips of our dataset. The emotion label bar and behavior label bar represent the labels of each frame for that video. Note that video number 31 has 1200 frames and video number 04 has 1040 frames totally

In Fig. 3 we demonstrate some frames of two sample video clips in our dataset along with both emotion and behavior ground-truth labels. As can be seen, the crowded videos might contain several behavior and emotion labels depending on individual’s temper and feeling in the scene. For instance, in video number 04, frames begin normally and individuals have neutral behaviors and feelings, but after a while (about 800 frames) gang of hoodlums attack the individuals and make them panicked and scared, and then all of them disperse.

**Fig. 4:**
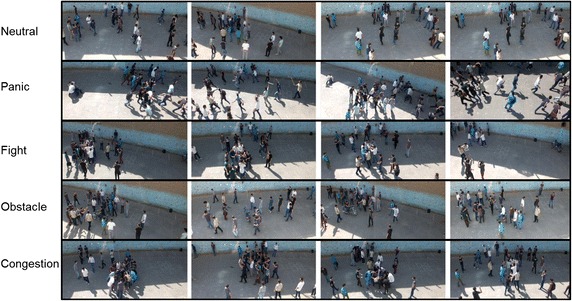
Example of different scenario clips. *Row 1*: four sample clips of neutral scenario. *Row 2*: four sample clips of panic scenario. *Row 3*: four sample clips of fight scenario. *Row 4*: four sample clips of obstacle (abnormal object) scenario. *Row 5*: four sample clips of congestion scenario

In Table 3, we annotate each crowd behavior type with its associated scenarios performed during dataset recording. Note that despite the presence of other scenarios, we tried to select more possible and realistic ones in our dataset. According to Table 3, it is obvious that *fight* is a sub level of *congestion* and suspicious backpack comes under two labels, namely *panic* and *Obstacle*. Some sample frames of our dataset along with their behavior type annotations are presented in Fig. 4.

**Table 3:** Crowd behavior types introduced in our dataset along with associated scenarios implemented for each type

Type of behavior	Scenarios
Panic	Suspicious backpack
	Hoodlum attack
	Earthquake
	Sniper attack
	Terrorist firework
Fight	Previous personal issues between individuals that suddenly meet each other in the crowd
	Intentional or unintentional bad physical contact between two or more people in the crowd
Congestion	Demonstration
	Helping out an individual facing Health problem
	Break up a fight between two or more individuals
Obstacle (abnormal object)	Bag theft with motorcycle
	Suspicious backpack
	An individual that fell to the ground for some reasons
	Motorcycle left in the crowd
	Motorcycle crossing the crowd
Neutral	Moving individuals with almost fixed velocity in random direction
	Two or more people meeting one another

For each crowd emotion type, some sample scenarios used in our dataset are presented in Table 4. The videos, the ground-truth annotations and the baseline codes will be available to public soon after publishing the paper. We believe this dataset can be used as a benchmark of future researches in both abnormal behavior detection and emotion recognition tasks.

**Table 4:** Crowd emotion types introduced in our dataset along with associated scenarios implemented for each type

Type of basic emotion	Scenarios
Angry	Previous personal issues between individuals suddenly meet each other
	Intentional or unintentional bad physical contact between two or more people
	Demonstration
	Motorcycle left in the crowd
	Motorcycle crossing the crowd
Happy	one or more individuals greeting in the crowd
Excited	Demonstration
	Excited bag theft with motorcycle
	Two or more friends suddenly visit each other in the crowd
Scared	Sniper attack
	Terrorist firework
	Hoodlum attack
	Motorcycle crossing the crowd
	Bag theft in the crowd with motorcycle
Sad	An individual facing health problem in the crowd
	Sad demonstration
Neutral	All videos begin with neutral frames

## Emotion-based crowd representation

We strongly believe that crowd behaviors are better explained by crowd attributes such as crowd emotion. So, instead of extracting low-level features and solve the classification problem by introducing a classifier that maps the feature vector to a specific class label, we explain how we indicate behavior classes with a set of crowd emotions. If we consider ground-truth emotion information available during both training and testing, we can simply regard them as part of input data and cope with a standard classification problem (see *Emotion*-*aware* baseline for evaluation, “Emotion-based representation experiments” section). However, if we don’t have emotion information during testing and only take them into account on the training data, the procedure becomes difficult to perform and the emotion information are not fully reliable. In this section, it is assumed that we have access to the emotion information only in training time.

Given a set of *N* video clips in the dataset$\left\{ {\left( {x^{(n)} ,e^{(n)} ,y^{(n)} } \right)} \right\}_{n = 1}^{N}$, we aim at learning a model wherein emotion labels *e* are used to assign a behavior label *y* to an unseen test video clip *x*. In training phase, each example is represented as a tuple (*f*, *e*, *y*) where *f* ∊ *F* ^*d*^ is the *d*-dimensional low-level feature extracted from video clip *x*. The behavior class label of the image is represented by *y* ∊ *Y* ^*d*^.

The crowd emotions of a video clip *x* are denoted by a *K*-dimensional vector *e* = (*e*_1_, *e*_2_, …, *e*_*k*_), where *e*_*k*_ ∊ *E*_*k*_(*k* = 1, 2, …, *K*) indicates the *K*th emotion of the video clip. For example, if the *K*th emotion attribute is “Angry”, we will have *E*_*k*_ = {0, 1}, where *e*_*k*_ = 1 means the crowd is “Angry”, while *e*_*k*_ = 0 means it is not. Since our dataset is designed to be applied also for standard multi-class emotion recognition setup, here, we describe each video clip with a binary-valued emotion attribute with a single non-zero value, i.e. *E*_*k*_ = {0, 1}(*k* = 1, 2, …, *K*), *s*.*t*. ‖*e*‖_0_ = 1. But we emphasize that our proposed method is not limited to binary-valued attributes with single emotion and simply can be extended to multi-emotion and continuous valued attributes.

Discarding the emotion information we can simply train a classifier *C*:*F* ^*d*^ → *Y*, which maps the feature vector *f* to a behavior class *y* (see low-level visual feature baseline for evaluation, “Baseline methods” section). On the contrary, by introducing the emotion attribute layer between the low-level features and behavior classes, the classifier *C* which maps *f* to a behavior class label, is decomposed into:

1 $$\begin{aligned} & H = B(\varepsilon (f)) \\ & \varepsilon :F^{d} \to E_{k} \\ & B:E_{k} \to Y \\ \end{aligned}$$

where *ɛ* includes *K* individual emotion classifiers $\{ C_{{e_{i} }} (f)\}_{i = 1}^{n}$, and each classifier maps *f* to the corresponding *i*th axis (emotion attribute) of *E* ^*n*^, *B* maps an emotion attribute *e* ∊ *E* ^*n*^ to a behavior class label *y* ∊ *Y*. The emotion classifiers are learned during training using the emotion annotations provided by our dataset. Particularly, the classifier $C_{{e_{i} }} (f)$ is a binary linear SVM trained by labeling the examples of all behavior classes whose emotion value *e*_*i*_ = 1 as positive examples and others as negative.

Assuming there is no emotion ground-truth information is available in test time, we represent each video clip *x* by *Φ*(*x*) ∊ *E*_*k*_:

2 $$\varPhi (x) = [s_{1} (x),s_{2} (x), \ldots ,s_{k} (x)]$$

where *s*_*k*_(*x*) is the confidence score of *K*th emotion classifier $C_{{e_{k} }}$ in *ɛ*. This *emotion*-*based crowd representation* vector has an entry for each emotion attribute and is used to show the degree of presence of an emotion attribute in a video clip (see “Emotion-based representation experiments” section). The mapping *B* is finally obtained by training a multi-class linear SVM for behavior classes on emotion-based crowd representation vectors. The fact that abnormal behavior classes and behavior instances share the same semantic space and the capability to manually define *B* make it possible to recognize a novel abnormal behavior class with no training samples available (Larochelle et al. 2008), which is out of the scope of current work.

### Emotions as latent variables

Given a set of *N* training instances $\left\{ {\left( {x^{(n)} , y^{(n)} } \right)} \right\}_{n = 1}^{N}$, we need to learn a classification model to recognize an unseen video clip *x*. As aforementioned, we select crowd emotions as attributes, which are discriminative and yet able to extract the intra-class changing of each behavior. Note that intra-class changing may cause video clips to correspond to different sets of emotion information, in spite of belonging to same behavior class. For instance, the behavior class *congestion* in some video clips of a dataset have the *angry* emotion attribute, while in other samples it might contain *happy* emotion attribute (see Fig. 1). Similar situation may happen for other behavior classes. To address this problem, emotion attributes are treated as latent variables and we learn the model using the latent SVM (Felzenszwalb et al. 2008; Wang and Mori 2009).

Regarding emotion attributes as an abstract part of a behavior class, we introduce a semantic space E^*n*^ where in location of an emotion attribute is defined as a latent variable, *e*_*i*_ ∊ E^*n*^. The probability of possessing this emotion attribute by a video clip is higher when we have the larger values of *e*_*i*_. Considering *W* as parameter vector, we aim at learning a classifier *f*_*W*_ to predict the behavior class of an unknown video clip *x* during testing, y* = arg max _*y*∊*Y*_ *f*_*W*_(*x*, *y*). Note that we cannot characterize this prediction by only the video-label pair (*x*, *y*) and its corresponding emotion-attribute values *e* ∊ E^*n*^ are also needed. Specifically, a video-label pair (*x*, *y*) is scored by the function of the following form:

3 $$f_{W} (x,y) = \arg \mathop {\hbox{max} }\limits_{{e \in {\rm E}}} \varphi (x,y,e)$$

where, *φ*(*x*, *y*, *e*) is a feature vector relating to raw feature *x*, a parameter vector preparing a weight for each feature *w*, and *y* is raw behavior class label for each feature. The linear model is defined as:

4 $$W^{T} \varphi (x,y,e) = W_{x} \varphi_{1} (x) + \sum\limits_{{l \in {\rm E}}} {\varphi_{1} (x) + } \sum\limits_{{l \in {\rm E}}} {W_{{e_{l} }}^{T} \varphi_{2} (x,e_{l} ) + \sum\limits_{{l,m \in {\rm E}}} {W_{{e_{l} ,e_{m} }}^{T} } } \varphi_{3} (e_{l} ,e_{m} )$$

where, parameter vector *W* is $W = \{ W_{x} ;W_{{e_{l} }} ;W_{{e_{l} ,e_{m} }} \}$, and E is an emotion attribute set.

In Eq. (4), if we only keep the potential function *W*_*x*_ *φ*_1_(*x*) and discard others, we can learn *W*_*x*_ by a binary linear SVM. By providing the score, the potential function *W*_*x*_ *φ*_1_(*x*) evaluates how well the raw feature *φ*_1_(*x*) of a video clip matches the model vector *W*_*x*_ which is a set of coefficients learned from the raw feature *x*. In our implementation, we use this observation and represent *φ*(*x*) as the score output of the pre-trained linear SVM instead of keeping it as a high-dimensional feature vector. As a result, *W*_*x*_ is a scalar value providing SVM score weights.

For a specific emotion attribute *e*_*l*_, the potential function $W_{{e_{l} }}^{T} \varphi_{2} (x,e_{l} )$ prepares the sore of an individual emotion attribute, and is used to show the presence of an emotion attribute in the video clip *x*. As we mentioned in “Emotion-based crowd representation” section, initial value of a specific emotion attribute *e*_*l*_ is extracted from its class label during training and is provided by a pre-trained emotion attribute classifier during testing. Simultaneous happening of pair of emotion attributes (*e*_*l*_, *e*_*m*_) are captured by edge function $W_{{e_{l} ,e_{m} }}^{T} \varphi_{3} (e_{l} ,e_{m} )$, in which the feature vector *φ*_3_(*e*_*l*_, *e*_*m*_) is a E × E dimensional indicator for edge function configurations and the corresponding $W_{{e_{l} ,e_{m} }}^{T}$ has weights of all configurations. From a set of training instances, the model vector *W* is learned by solving the following formulation as learning objective function:

5 $$W^{ * } = \mathop {\hbox{min} }\limits_{w} \lambda \left\| W \right\|^{2} + \sum\limits_{j = 1}^{n} {\hbox{max} (0,1 - y_{j} \cdot f_{w} (x_{j} ))}$$

where, *λ* is the trade-off parameter controlling the amount of regularization, and the second term performs a soft-margin. Due to the existence of inner max in *f*_*W*_, the objective function in Eq. (5) is semi-convex. In our implementation, the optimization problem is solved by adopting the coordinate descent (Felzenszwalb et al. 2008), as follows:

- Holding *W* fixed, we find the best emotion attribute configuration e* that maximizes *W*.*φ*(*x*, *y*, *e*).
- Holding *e* ^*^ fixed, we find parameters *W* that optimizes convex objective in Eq. (5).

In our current process, we use training data for learning Emotion attribute relation graph. For the sake of computational efficiency, we dedicate two statues, namely ({0} and {1}) to emotion attributes. Finally, we apply belief propagation (Felzenszwalb et al. 2008) to find the best emotion attribute configuration *e* ^*^ for *f*_*W*_(*x*, *y*) = max _*e*∊E_ *W* ^*T*^.*φ*(*x*, *y*, *e*) (see latent-emotion crowd representation experiment, “Emotion-based representation experiments” section).

## Experiments

**Fig. 5:**
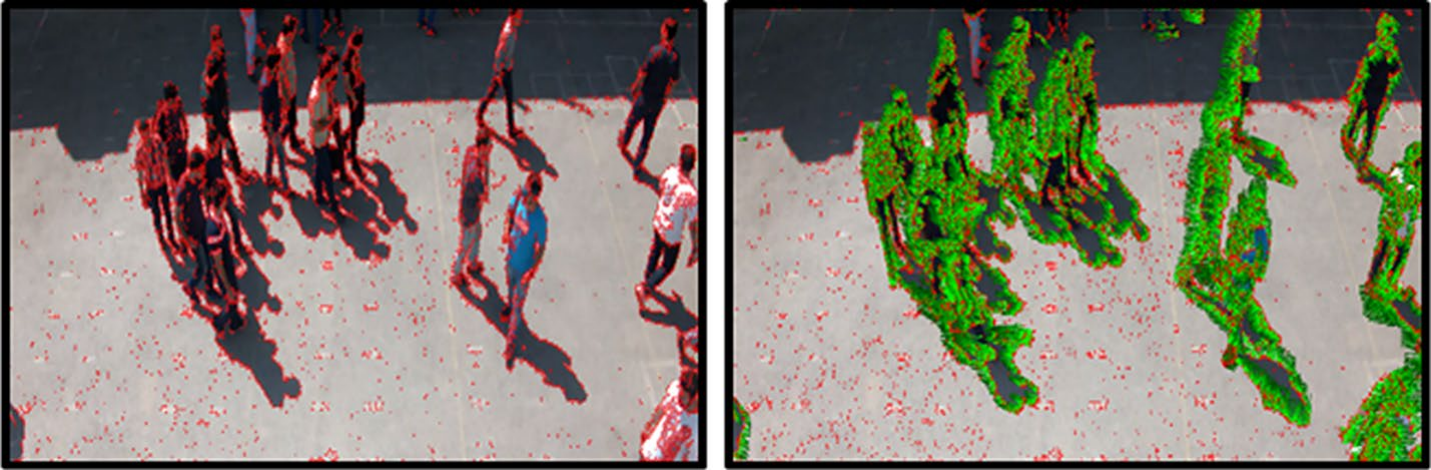
Dense trajectories computed for different crowded scenarios in our dataset. *Red marks* are the end points of the trajectories

**Fig. 6:**
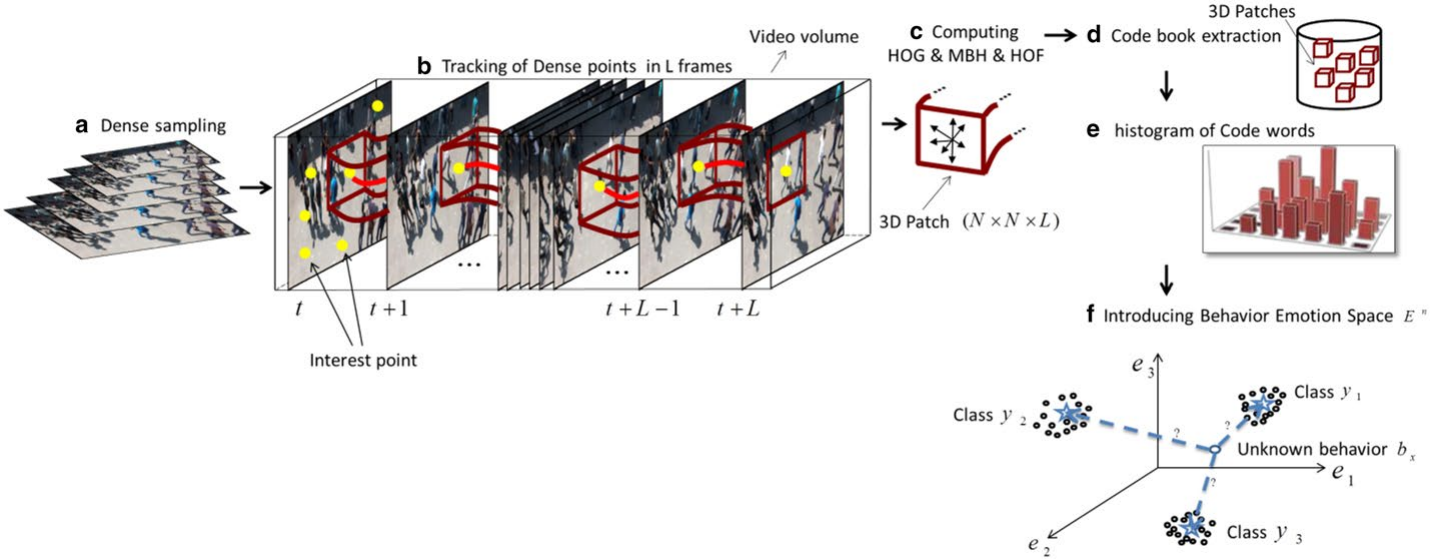
Mixture of low-level features namely, HOG, HOF and MBH with high-level semantic concepts (emotion labels) for the task of crowd behavior classification

The broad variety of crowd emotion attribute needs a low-level feature representation to explain several visual aspects. In this section we first apply state-of-the-art dense trajectories (Wang et al. 2011, 2012) approach for behavior recognition as a baseline. Figure 5 shows the dense trajectories computed for different crowded scenarios in our dataset. Following that, we propose emotion-based crowd representation by introducing crowd emotions as intermediate representations for the type of behavior classification. We believe that by applying emotion layer as a bridge between low-level features and crowd behavior labels, it is possible to construct a more efficient learning behavior classification framework. In Fig. 6, the schematic form of applied baseline is demonstrated.

Note that we fixed the evaluation protocol during all the experiments. Furthermore, we divide the train and test data in a leave-one-sequence-out fashion. More precisely, for 31 iterations (equal to number of video sequences) we leave all the video clips of a sequence out for test and train on all video clips of 30 remaining sequences. In evaluation process, we used the average accuracy criterion both in tables and confusion matrices. We use dense trajectory features for all confusion matrices.

### Baseline methods

#### Low-level visual feature baseline

We adopt the well-known dense trajectories (Wang et al. 2011, 2012) to represent each video clip of our dataset. For this purpose, the state-of-the-art trajectory-aligned descriptors, namely histogram of oriented gradients (HOG) (Dalal and Triggs 2005), histogram of optical flow (HOF) (Laptev et al. 2008), motion boundary histogram (MBH) (Dalal et al. 2006) and dense trajectories (Wang et al. 2011) are computed within a space–time volume around the trajectory to encode the motion information. The size of the volume is 32 × 32 pixels and 15 frames long (see Fig. 6).

In order to evaluate our dense trajectory features, we use a standard bag-of-features approach. More specifically, we first construct a codebook for each descriptor (HOG, HOF, MBH, and Trajectory) separately. The number of visual words per descriptor is fixed to *d* = 1000 which has shown to empirically yield good results over a wide range of datasets. For the sake of time and simplicity, we cluster a subset of 150,000 randomly selected training features using k-means. In order to increase precision, k-means is initialized for 10 times and the result with the lowest error is kept. Descriptors are assigned to their closest vocabulary word using Euclidean distance. The resulting histograms of visual word occurrences are used as video descriptors.

For classification a standard one-vs-all multi-class SVM classifier is used. For this purpose, we separately evaluate computed low-level feature descriptors using only the crowd behavior ground-truth label information. The average precision for each of them is reported in first column of Table 5. Results show that dense trajectory feature achieved 38.71 % precision in crowd behavior type detection and has better performance compared to the other four feature descriptors. Table 6 shows the confusion matrix for five different crowd behavior classes with varied performance. Some interesting observations can be made from the confusion tables. For example, the *Panic* crowd behavior class has the best average precision of 74.82 % compared to other classes, probably because of being a simpler visual task.

**Table 5:** Comparison of dense trajectory descriptor on low-level visual features, emotion-aware and emotion-based categories in our dataset

Our dataset			
	Low-level visual feature	Emotion-aware	Emotion-based
Dense trajectory	38.71	83.79	43.64

We report average accuracy over all classes for our dataset

**Table 6:** Confusion matrix for each low-level visual feature class

Truth	Prediction				
	Panic (%)	Fight (%)	Congestion (%)	Obstacle (%)	Neutral (%)
Panic	74.82	15.18	5.64	3.39	0.97
Fight	24.489	30.47	17.18	18.24	9.63
Congestion	32.17	18.11	23.43	18.91	7.38
Obstacle	9.25	25.54	19.02	27.94	18.25
Neutral	9.40	16.80	17.65	19.27	36.88

Also, some *Panic* crowd behavior classes are misclassified as *fight* with the most average precision since both classes share similar motion patterns (very sharp movements).

### Emotion-based representation experiments

In this part we present a series of experiments with respect to our emotion-based proposed method described in “Emotion-based crowd representation” section. For this purpose, we first assume that we have access to the ground-truth emotion labels during both testing and training. Unlike, for the second experiment it is assumed that we have access to them only during training.

#### Emotion-aware baseline

In this part we use the basic ground-truth crowd emotion information, namely “*angry*”, “*happy*”, “*excited*”, “*scared*”, “*sad*” and “*neutral*”, respectively to create attribute features. We first simply build a 6 dimension binary feature vector for all test and train data. As an example, if the crowd possesses “happy” emotion class, the feature vector can be represented as{0, 1, 0, 0, 0, 0} ∊ *E* ^6^, with each dimension indicating the presence or absence of a crowd emotion attribute class. As next process, considering created features, we train a multi-class SVM classifier using crowd behavior labels.

During testing we evaluate the pre-trained classifier with test examples. The average precision result for this experiment over all crowd behavior classes is presented in second column of Table 5.

Such significant margins suggest that having a precise emotion recognition method can be so helpful for crowd behavior understanding. Inspired by this result, in the following experiments we employ the emotion as mid-level representation for crowd behavior representation.

#### Emotion-based crowd representation experiment

In this part, we first simply use the ground-truth emotion information to separately evaluate the aforementioned low-level feature descriptors. Table 7 shows the performance comparison between varied combinations of different types of emotion information in confusion matrix based on dense trajectory feature descriptors with average accuracy of 34.13 %. The results reported in the confusion matrix of Table 7 can fairly be used for abnormality behavior detection procedure. As the second part of this experiment, we assume that there is no emotion information available for the test data, so we learn a set of binary linear SVMs using emotion labels of training data. As we mentioned in “Emotion-based crowd representation” section, we know $C_{{e_{i} }} (f)$ as emotion classifiers. The output of emotion classifiers is a vector wherein each dimension shows the confidence score of each emotion attribute prediction. We consider this vector as an emotion-based crowd representation vector for behavior classification. We extract this vector for all the train and test data and following that, train a multi-class SVMs with behavior labels. This behavior classifier is finally evaluated on test data to report the final accuracy of behavior classification.

**Table 7:** Confusion matrix for six predefined emotion classes

Truth	Prediction					
	Angry (%)	Happy (%)	Excited (%)	Scared (%)	Sad (%)	Neutral (%)
Angry	25.42	15.40	16.12	26.45	11.14	5.47
Happy	17.60	18.10	23.92	15.05	19.06	6.27
Excited	20.39	11.90	32.22	5.91	16.11	13.47
Scared	14.02	10.22	6.58	65.92	2.86	0.40
Sad	26.92	6.75	6.31	27.66	29.56	2.80
Neutral	9.59	17.88	17.51	7.54	13.90	33.58

We applied this method separately to HOG, HOF, MBH, Trajectory and Dense trajectory low-level feature descriptors. The average accuracy resulting for each of them is presented in second column of Table 8. As can be seen, dense trajectory feature achieved the best precision with 43.64 % among the other low level features. This experiment has the highest accuracy compared to two other baselines by increasing them almost 7 percentage points Also in confusion matrix in Table 9, the best detection result belongs to “*Panic*” behavior class with 71.87 % and the most conflict to this class belongs to “*fight*” behavior category with 11.88 %. On the other hand, the worst detection result belongs to “congestion” behavior class with the most conflict of 21.92 % to “*panic*” behavior class. These results are in line with the average accuracies achieved in emotion based classifiers and emotion-aware baseline. They also support the idea of having better emotion recognition classifiers and more precise emotion labels, boost the performance of crowd behavior category recognition.

**Table 8:** Comparison of different feature descriptors (trajectory, HOG, HOF, MBH and dense trajectory) on low level visual feature, emotion-based and latent-emotion categories in our dataset

	Our dataset		
	Low-level visual feature	Emotion-based	Latent-emotion
Trajectory	35.30	40.05	40.04
HOG	38.80	38.77	42.18
HOF	37.69	41.50	41.51
MBH	38.53	42.72	42.92
Dense trajectory	38.71	43.64	43.90

We report average accuracy over all classes for our dataset

**Table 9:** Confusion matrix for each emotion-based class

Truth	Prediction				
	Panic (%)	Fight (%)	Congestion (%)	Obstacle (%)	Neutral (%)
Panic	71.87	11.88	7.49	4.64	4.19
Fight	21.72	34.37	13.24	18.76	11.91
Congestion	21.92	18.98	30.66	18.69	9.75
Obstacle	11.01	20.11	13.86	33.19	21.83
Neutral	10.11	12.67	8.46	20.65	48.11

#### Latent-emotion crowd representation experiment

Finally, as we mentioned in “Emotion-based crowd representation” section, we treat emotion labels as latent variables, and learn the model using the latent SVM. In third column of Table 8, the result of this experiment is reported which is 43.9 % for dense trajectory and is the best result compared to other results. Considering Table 8, it is obvious that the result for *latent*-*emotion* experiment is much better compared to *low*-*level visual feature* experiment and is better compared to *emotion*-*based* experiment.

## Conclusion

In this paper, we proposed a novel crowd dataset with both the crowd emotion and behavior annotations. We believe this dataset not only can be used as a benchmark in computer vision community, but also can open up doors toward understanding the correlations between the two tasks of “crowd behavior understanding’’ and “crowd emotion recognition’’. We have proposed to represent human behaviors by a set of intermediate concepts called emotion attributes which are either manually specified or learnt from training data. We have introduced a unified framework wherein the emotion attributes can be effectively chosen in a discriminative fashion. Extensive experiments have been adopted to validate our claims and have confirmed our intuition that an emotion attribute-based crowd representation is a critical building block for modeling complex behaviors from videos. In particular, future work will be directed towards recognizing a novel behavior class with no training samples available, by manually defining the emotion-to-behavior mapping function. We will also perform our experiments with some large crowd data sets to validate the proposed methodology in more effective manner.
