# Erratum to: Crowd behavior representation: an attribute-based approach

**DOI:** 10.1186/s40064-016-3754-4

**Published:** 2016-12-30

**Authors:** Hamidreza Rabiee, Javad Haddadnia, Hossein Mousavi, Moin Nabi, Vittorio Murino, Nicu Sebe

**Affiliations:** 1 Electrical Engineering Department, Hakim Sabzevari University, Sabzevar, Iran; 2 Biomedical Engineering Department, Hakim Sabzevari University, Sabzevar, Iran; 3 Pattern Analysis and Computer Vision Department (PAVIS), Istituto Italiano di Tecnologia, Genoa, Italy; 4 DISI, University of Trento, Trento, Italy

## Erratum to: SpringerPlus (2016) 5:1179 DOI 10.1186/s40064-016-2786-0

After publication of this article (Rabiee et al. 2016), we noticed that some of the authors were inadvertently omitted from the author list. These authors, Moin Nabi, Vittorio Murino and Nicu Sebe, have been included in the corrected author list above. Additionally, the images in Fig. 1 have been removed from this article as we did not obtain the necessary permissions to publish them. This does not affect the scientific validity of the article.

## References

[CR1] Rabiee H, Haddadnia J, Mousavi H, Nabi M, Murino V, Sebe N (2016). Crowd behavior representation: an attribute-based approach. Springerplus.

